# Impact of cytomegalovirus reactivation on clinical outcomes in immunocompetent critically ill patients: protocol for a systematic review and meta-analysis

**DOI:** 10.1186/s13643-016-0303-8

**Published:** 2016-07-28

**Authors:** Philippe Lachance, Justin Chen, Robin Featherstone, Wendy Sligl

**Affiliations:** 1 Division of Critical Care Medicine, Faculty of Medicine and Dentistry, University of Alberta, 2-124 Clinical Sciences Building, 8440 – 112th Street, Edmonton, Alberta T6G 2B7 Canada; 2 Division of Infectious Diseases, Faculty of Medicine and Dentistry, University of Alberta, Edmonton, Canada; 3 Alberta Research Centre for Health Evidence (ARCHE), Department of Pediatrics, University of Alberta, Edmonton, Canada

**Keywords:** Cytomegalovirus, Intensive care unit, Meta-analysis, Systematic review, Protocol

## Abstract

**Background:**

Cytomegalovirus (CMV) reactivation in critically ill patients is a well-recognized phenomenon with an incidence as high as 71 %. A number of studies have investigated the association between CMV reactivation and outcomes in critically ill patients with conflicting results. We propose to conduct a systematic review and meta-analysis to determine the impact of CMV reactivation on patient-centered outcomes and measures of health resource utilization in immunocompetent critically ill patients.

**Methods:**

In consultation with a research librarian, a search strategy will be developed and electronic databases (i.e., Ovid MEDLINE, Ovid EMBASE, and the Cochrane Library including the Cochrane Database of Systematic Reviews, the Cochrane Central Register of Controlled Trials (CENTRAL)) will be searched for original studies. Selected grey literature sources will be hand-searched. Search themes will include cytomegalovirus, intensive care unit, and sepsis. Citation screening, selection, quality assessment, and data abstraction will be performed in duplicate. Pooled effect estimates of the impact of CMV reactivation on selected patient-centered outcomes and measures of health resource utilization will be described.

**Discussion:**

This systematic review aims to explore the impact of CMV reactivation on patient-centered outcomes and health resource utilization in immunocompetent critically ill patients. Our results will help to better define the burden of disease associated with CMV reactivation. Indeed, evidence to date suggests increased mortality in this patient population. However, the relationship between CMV reactivation and health resource utilization remains less clear. Based on our results, future study on the impact of CMV treatment or prophylaxis on outcomes (including those other than mortality) may be warranted.

**Systematic review registration:**

PROSPERO CRD42016035446

**Electronic supplementary material:**

The online version of this article (doi:10.1186/s13643-016-0303-8) contains supplementary material, which is available to authorized users.

## Background

It is estimated that 40 to 100 % of immunocompetent adults are cytomegalovirus (CMV) seropositive globally [1,2]. In Canada, seroprevalence ranges between 60 and 80 % [2]. Most primary infections occur in childhood and are subclinical or present with non-specific symptoms. CMV subsequently remains latent in monocytes and macrophages [3, 4]. This state of latency allows CMV to reactivate when host defenses become compromised, such as in critical illness. CMV reactivation in critically ill patients is well-recognized with as high as 71 % incidence [5]. The consequences of CMV reactivation in immunocompromised patient populations, such as solid organ transplantation, have been well described [6]. However, the clinical significance in immunocompetent patients remains controversial. Some postulate viral pathogenesis by direct cytopathic effect (tissue-invasive disease) [5, 7–9], by an over-response of the immune system [5, 10] or by inactivation of host defenses allowing opportunistic pathogens to establish infection [5, 11, 12]. Others have suggested that CMV reactivation is only a marker of illness severity [5].

Since the 1990s, a number of studies have investigated the association between CMV reactivation and outcomes in critically ill patients. In 1990, Domart et al. examined patients with mediastinitis following cardiac surgery who were CMV infected, defined by blood and/or urine viral cultures. They showed a significant increase in mortality and hospital length of stay compared with CMV-uninfected patients [13]. Thereafter, other studies have also reported increased mortality [14, 15], increased duration of mechanical ventilation [11, 12], increased length of intensive care unit (ICU) stay [16, 17], and increased incidence of nosocomial infections [18]. Contrasting this data, Heininger et al. failed to demonstrate a difference in in-hospital mortality in patients with CMV DNAemia [19]. More recently, Frantzeskaki et al. came to the same conclusion [20].

With a growing number of studies examining the impact of CMV reactivation on outcomes and discrepancies in the available data, systematic reviews and meta-analyses have been previously undertaken. In 2009, Osawa et al. conducted the first systematic review on the subject, which included 13 studies. Four studies reported data on duration of mechanical ventilation—all of which showed a statistically significant negative effect of CMV reactivation on this outcome. No pooled estimate was available, as they did not perform a meta-analysis. All but two of the included studies reporting death showed no difference between CMV positive and negative patients and mortality [21]. Conversely, Kalil et al. published a meta-analysis the same year including eight studies and 633 patients showing a twofold increase in the odds ratio of death with CMV infection. There was however no data on other clinical outcomes [22]. These authors updated their results after Heininger et al. published the study mentioned above showing no difference in mortality [19]. The effect of CMV infection on mortality remained significant [23]. Finally, Coisel et al. performed a prospective study on the prognosis of CMV-infected mechanically ventilated patients in which they included a meta-analysis demonstrating a positive association between CMV antigenemia and mortality [24]. Since the publication of the last meta-analysis, at least four additional studies have been published on this topic with varying results [20, 25–27].

Considering the availability of new evidence and the absence of meta-analyses examining important outcomes such as mechanical ventilation duration, ICU length of stay, or incidence of nosocomial infection, we propose to conduct a systematic review and meta-analysis to determine the impact of CMV reactivation on various clinical outcomes in immunocompetent critically ill patients.

## Objectives

The aim of our systematic review is to determine the impact of CMV reactivation (defined by either pp65 CMV antigenemia or blood/plasma CMV DNAemia detected by quantitative nucleic acid testing [NAT]) compared to no reactivation on patient-centered outcomes (including mortality, duration of mechanical ventilation, nosocomial infection) or health services utilization (ICU length of stay, hospital length of stay) in immunocompetent critically ill patients.

## Methods

### Study design

A systematic review will be performed using guidelines from The Cochrane Collaboration and Center for Reviews and Dissemination and described according to PRISMA-P guidelines (available at: http://www.systematicreviewsjournal.com/content/4/1/1) (see Additional file 1) [28].

### Study registration

In accordance with PRISMA-P guidelines, our systematic review will be registered with the International Prospective Register of Systematic Reviews (PROSPERO) (www.crd.york.ac.uk/prospero; registration number CRD42016035446).

### Criteria for considering studies for this review

Inclusion criteria:

1. Population: We will include studies of adults [>18 years of age] who are immunocompetent [i.e., we will specifically exclude solid organ or bone marrow transplant patients, those with advanced HIV/AIDS, or those receiving cytotoxic therapies] admitted to any type of ICU for any cause. Patients with documented CMV tissue invasive disease will also be excluded.
2. Intervention: Critically ill patients with CMV reactivation, defined by either pp65 CMV antigenemia or blood/plasma DNAemia, will be compared to those without reactivation.
3. Outcomes: We will examine the impact of CMV reactivation on at least one patient-centered outcome or measure of health resource utilization.
4. Design: We will include observational studies (prospective and retrospective) as well as randomized trials. We will exclude case reports and case series.

### Exclusion criteria

Studies will be excluded if they do not fulfill all of the inclusion criteria; if they are published in a language other than English or French, use serology to define CMV reactivation, or include immunocompromised patients (as defined above).

### Search methods for identification of studies

PROSPERO (http://www.crd.york.ac.uk/prospero) was searched for any registered systematic reviews on this topic (November 30, 2015).

The search strategy was developed in consultation with an expert librarian/information specialist at the Alberta Research Centre for Health Evidence (ARCHE) at the University of Alberta and has undergone subsequent peer-review by a second specialized librarian using the Peer Review of Electronic Search Strategies checklist [29]. The information specialist will search electronic databases: Ovid MEDLINE, Ovid EMBASE, and the Cochrane Library including the Cochrane Database of Systematic Reviews, the Cochrane Central Register of Controlled Trials (CENTRAL) for three domains: cytomegalovirus, intensive care unit, and sepsis. Database search results will be restricted to papers published in English or French language and published after 1990 for screening.

Appropriate truncation and wildcards will be used in the search to account for plurals and/or variations in the spelling of search terms (see Additional file 2 for example of the search strategy in Medline). Bibliographic records will be exported to EndNote X7 (Thomson Reuters, Philadelphia, Pennsylvania) for screening. Additional sources will be included in the search strategy. The cited and citing references of selected key studies will be searched for relevant articles. Grey literature sources will be searched. We will identify and search relevant conference proceedings from the past 2 years: *Infectious Diseases Society of America (IDSA) IDWeek, Canadian Association for Clinical Medical Microbiology and Infectious Diseases-Association of Medical Microbiology and Infectious Diseases Canada (CACMID-AMMI) Annual Meeting, European Society of Clinical Microbiology and Infectious Diseases (ESCMID) Annual Congress, Society of Critical Care Medicine Annual Congress, International Symposium on Intensive Care and Emergency Medicine.* We will also search the trial registry at www.clinicaltrials.gov for trials conducted during the past 2 years.

### Study selection

Potentially eligible articles will be identified by two authors after independent review of the titles and abstracts of all articles identified by the search. The full text of all articles deemed potentially relevant will be independently reviewed, again by the two authors, for inclusion using pre-defined eligibility criteria. Any disagreements that arise will be resolved through discussion and/or arbitration by the senior author. Inter-rater agreement will be calculated.

### Data extraction

Data will be abstracted from relevant studies using a standardized electronic data collection form (Additional file 3). Data extracted will include publication-related information, patient-related information (demographic characteristics and medical comorbidities, design, and quality assessments of the included studies, inclusion, and exclusion criteria), and the method of CMV detection. Patient-related outcomes and health service use will also be collected. This form will undergo pilot testing. Abstraction will be performed in duplicate by the same two authors. Any disagreements that arise will be resolved through discussion or arbitration by the senior author. The authors of the retrieved studies and/or documents will be contacted for further information as necessary.

Study methodological quality will be rated using the Newcastle-Ottawa Scale (NOS) [30] for observational studies and the Cochrane Collaboration’s tool [31] for trials.

### Outcomes

The primary outcome of our study will be mortality (however defined in the included studies). Secondary outcomes will be mechanical ventilation duration, nosocomial infections, need for renal replacement therapy, ICU length of stay, and hospital length of stay.

### Analysis

Pooled effect estimates of the impact of CMV reactivation on patient-centered outcomes and health service use will be reported. We will assess and quantify statistical heterogeneity for each pooled summary estimate using Cochran’s Q statistic and the *I*^2^ statistic, respectively [32]. Pooled analysis will be performed using random effects models and reported as odds ratios with 95 % confidence intervals for categorical variables and weighted mean differences with 95 % confidence intervals for continuous variables, respectively. We expect to see heterogeneity as a result of different CMV detection methods, varied study designs, and due to the evolution in ICU care over time. To address this, we plan to perform a number of pre-defined sensitivity analyses according to the following variables: study design (observational vs. RCT), year of study (before or after 2005), and studies including only mechanically ventilated patients. Publication bias will be assessed using Egger’s regression models and visualized using funnel plots [33]. All analyses will be performed using RevMan statistical software.

### Expected limitations

Based on screening of the literature, we expect some degree of heterogeneity in our study populations and in the frequency of CMV monitoring. The latter may affect our ability to detect CMV reactivation. Heterogeneity may limit the interpretation of our results.

Inter-laboratory CMV viral loads using laboratory-developed NATs can vary significantly—especially at low viral loads (from 2 log_10_ copies/mL to 4.3 log_10_ copies/mL on the same specimen) and when testing was performed prior to the development of WHO International Calibration Standards in 2010 [34]. We expect that this will have minimal impact on our results as we will use any CMV antigenemia or DNAemia to define CMV reactivation.

## Discussion

This systematic review and meta-analysis will explore the association between CMV reactivation, patient-centered outcomes, and health resource utilization in immunocompetent critically ill patients. Our results will help to better define the burden of disease associated with CMV reactivation. Indeed, evidence to date suggests increased mortality in this patient population. However, the relationship between CMV reactivation and health resource utilization is less clear. Based on our results, future study on the impact of CMV treatment or prophylaxis on outcomes (including those other than mortality) may be warranted.

## Abbreviations

AIDS, acquired immunodeficiency syndrome; ARCHE, Alberta Research Centre for Health Evidence; CACMID-AMMI, Canadian Association for Clinical Medical Microbiology and Infectious Diseases-Association of Medical Microbiology and Infectious Diseases Canada; CENTRAL, Cochrane Central Register of Controlled Trials; CMV, cytomegalovirus; DNA, deoxyribonucleic acid; ESCMID, European Society of Clinical Microbiology and Infectious Diseases; HIV, human immunodeficiency virus; ICU, intensive care unit; IDSA, Infectious Diseases Society of America; NAT, nuclear acid testing; RCT, randomized control trial

## Additional files

Additional file 1: Preferred Reporting Items for Systematic review and Meta-Analysis Protocols (PRISMA-P) 2015 checklist: recommended items to address in a systematic review protocol. (DOC 120 kb) Additional file 2: Example of the search strategy in Medline. ( DOC 113 kb) Additional file 3: Data to be collected. (DOC 99.7 kb)
