# Supplementary material for: Impact of cytomegalovirus reactivation on clinical outcomes in immunocompetent critically ill patients: protocol for a systematic review and meta-analysis
Source: Syst Rev. 2016 Jul 28;5:127. doi: 10.1186/s13643-016-0303-8 (PMC4963995; doi:10.1186/s13643-016-0303-8)
Supplement: Additional file 2: — Example of the search strategy in Medline. ( DOC 113 kb) [file 13643_2016_303_MOESM2_ESM.docx]

**Additional file 2. Example of the search strategy in Medline**

1. Cytomegalovirus/

2. Cytomegalovirus Infections/

3. CMV*.tw,kf.

4. cytomegal*.tw,kf.

5. (herpes virus 5 or herpesvirus 5).tw,kf.

6. (herpes virus five or herpesvirus five).tw,kf.

7. (HHV 5 or HHV five).tw,kf.

8. (salivary gland* adj2 virus*).tw,kf.

9. or/1-8 [Combined MeSH & text words for CMV]

10. Coronary Care Units/

11. Critical Care/

12. Critical Illness/

13. Intensive Care Units/

14. Multiple Organ Failure/

15. Respiration, Artificial/

16. Respiratory Care Units/

17. exp Sepsis/

18. ((artificial* or mechanic*) adj (respirat* or ventilat*)).tw,kf.

19. bacteremia*.tw,kf.

20. (blood* adj2 poison*).tw,kf.

21. candidemia*.tw,kf.

22. (coronary care adj (department* or unit* or ward or wards)).tw,kf.

23. critical care.tw,kf.

24. (critical* adj2 (department* or unit* or ward or wards)).tw,kf.

25. critical* ill*.tw,kf.

26. endotoxemia*.tw,kf.

27. ((endotoxic or toxic) adj shock*).tw.

28. fungemia*.tw,kf.

29. intensive care*.tw,kf.

30. (intensive adj2 (department* or unit* or ward or wards)).tw,kf.

31. intensivist*.tw,kf.

32. (ICU* or SICU*).tw,kf.

33. (multi* organ adj (disfunction* or dis function* or dysfunction* or dys function* or failure*)).tw,kf.

34. (multi* system adj (disfunction* or dis function* or dysfunction* or dys function* or failure*)).tw,kf.

35. (py?emia* or pyohemia* or sepsis or septic*).tw,kf.

36. (respiratory care adj (department* or unit* or ward or wards)).tw,kf.

37. viremia*.tw,kf.

38. or/10-37 [Combined MeSH & text words for ICU]

39. and/9,38 [Combined CMV & ICU concept searches]

40. animals/ not (animals/ and humans/)

41. 39 not 40 [Human studies filter]

42. (adolescen* or babies or baby or birth* or child* or infan* or neonat* or NICU* or new born* or newborn* or p?ediatric* or PICU* or prematur* or postmatur* or teen*).ti.

43. 41 not 42 [Excluded pediatric studies]

44. transplant*.ti.

45. 43 not 44 [Excluded transplant studies]

**Additional file 2. Example of the search strategy in Medline (continued)**

46. (case reports or comment or editorial or letter).pt.

47. 45 not 46 [Excluded publication types]

48. limit 47 to (english or french) [Language limit]

49. limit 48 to yr="1990-current" [Publication date limit]

50. remove duplicates from 49
