# Supplementary material for: Impact of cytomegalovirus reactivation on clinical outcomes in immunocompetent critically ill patients: protocol for a systematic review and meta-analysis
Source: Syst Rev. 2016 Jul 28;5:127. doi: 10.1186/s13643-016-0303-8 (PMC4963995; doi:10.1186/s13643-016-0303-8)
Supplement: Additional file 3: — Data to be collected. (DOC 99.7 kb) [file 13643_2016_303_MOESM3_ESM.docx]

**Additional file 3.** **Data to be collected**

| **Data** | **Description** |
| --- | --- |
| Author | First author name |
| Reference | Journal, Issue, volume, pages |
| Country | Country in which the study was conducted |
| Year | Start and end date of study |
| Design | Observational (prospective vs. retrospective), RCT |
| Control group | Description of control group (historical, etc.) |
| Number of centers | Single-center, multi-center |
| Population | Restricted to mechanically ventilated patients or not |
| Type of ICU | Medical, surgical or mix |
| Inclusion criteria | List all inclusion criteria of the study |
| Method of CMV detection (even if mentioned in inclusion criteria) | pp65 antigenemia assay, NAT or both |
| Frequency of monitoring |  |
| Exclusion criteria | List all exclusion criteria of the study |
| Study Quality | See Appendix 2 |
| Funding | Industry vs. publicly funded |
| **Outcomes** | **Clinical outcomes** |
| Mechanical ventilation [duration , ventilator free days (however defined)] | Yes or no |
| Nosocomial infection [however defined] | Yes or no |
| Death [ICU, 7-day, hospital, 30-day, long-term [however defined]] | Yes or no |

**Additional file 3.** Data to be collected (continued)

| **Data** | **Description** |
| --- | --- |
| **Health resources utilization** |  |
| ICU length of stay | Number of days |
| Hospital length of stay | Number of days |
| **Results** |  |
| CMV incidence |  |
| Number | Total number of patients included |
| Age | Mean [SD] |
| Gender | % male |
| Baseline CMV serostatus | % positive in each group |
| Duration of illness before ICU | Days |
| APACHE II score | Number |
| SOFA score | Number |
| Creatinine | umol/L |
| PaO2/FiO2 |  |
| Mechanical ventilation | Number |
| MAP | mmHg |
| Dose of noradrenaline | mcg/kg/min |
| Clinical outcomes | All values related to clinical outcomes will be entered separately in our meta-analysis |
| Health services use | All values related to health services use will be entered separately in our meta-analysis |

List of abbreviations: CMV : Cytomegalovirus, ICU : Intensive Care Unit, MAP : Mean Arterial Pressure, NAT : Nuclear Acid Testing, RCT : Randomized Control Trial, SD: Standard Deviation
